# Thermoresponsive Nanocellulose Films as an Optical Modulation Device: Proof-of-Concept

**DOI:** 10.1021/acsami.1c03541

**Published:** 2021-05-19

**Authors:** Aayush Kumar Jaiswal, Ari Hokkanen, Vinay Kumar, Tapio Mäkelä, Ali Harlin, Hannes Orelma

**Affiliations:** † Biomass Processing and Products, VTT Technical Research Centre of Finland Ltd., Tietotie 4E, 02044 Espoo, Finland; ‡ Microelectronics, VTT Technical Research Centre of Finland Ltd., Tietotie 3, 02044 Espoo, Finland; § Sensing and Integration, VTT Technical Research Centre of Finland Ltd., Tietotie 3, 02044 Espoo, Finland

**Keywords:** nanocellulose, optical films, thermochromic, hybrid material, optical modulation

## Abstract

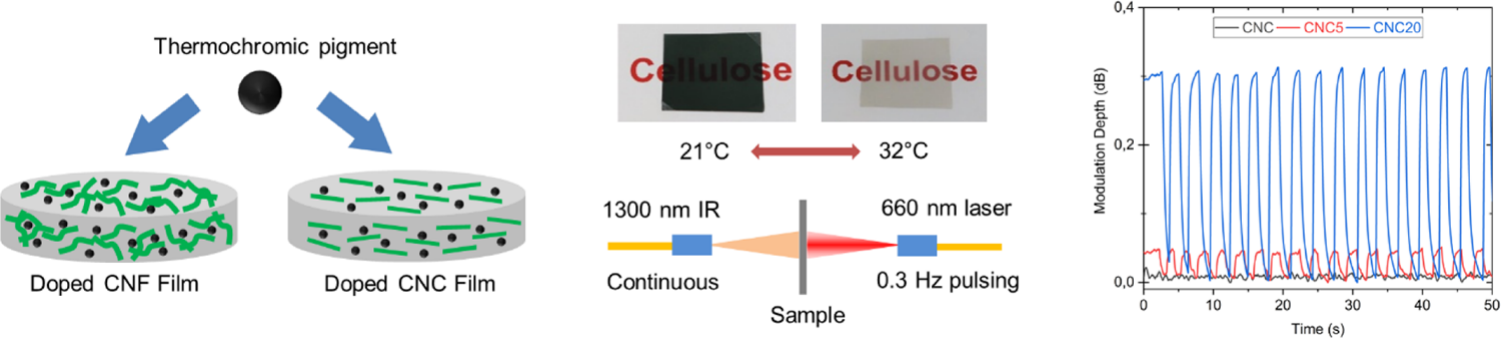

Flexible optoelectronic technologies are becoming increasingly important with the advent of concepts such as smart-built environments and wearable systems, where they have found applications in displays, sensing, healthcare, and energy harvesting. Parallelly, there is also a need to make these innovations environmentally sustainable by design. In the present work, we employ nanocellulose and its excellent film-forming properties as a basis to develop a green flexible photonic device for sensing applications. Cellulose nanofibrils (CNFs) and cellulose nanocrystals (CNCs) were used as matrix materials along with a black thermochromic pigment to prepare thermoresponsive hybrid films. Optical properties of nanocellulose films such as transparency and haze were tuned by varying pigment loading. Nearly 90% transparent CNF and CNC films could be tuned to reduce the transmission to as low as 4 and 17%, respectively. However, the films regained transparency to up to 60% when heated above the thermochromic transition temperature (31 °C). The thermoresponsive behavior of the prepared films was exploited to demonstrate an all-optical modulation device. Continuous infrared light (1300 nm) was modulated by using a 660 nm visible diode laser. The laser intensity was sufficient to cause a localized thermochromic transition in the films. The laser was pulsed at 0.3 Hz and a uniform cyclic modulation depth of 0.3 dB was achieved. The demonstrated application of functional nanocellulose hybrid films as a light switch (modulator) could be harnessed in various thermally stimulated sensing systems such as temperature monitoring, energy-saving, and anti-counterfeiting.

## Introduction

The demand for flexible electronic and photonic technologies is arising with the introduction of revolutionary concepts such as smart-built environments and wearable systems. Flexible electronic devices have found applications in displays, sensing, transistors, antennas, healthcare, energy storage, and harvesting.^1^

Flexible optoelectronics typically utilizes ultrathin glass, metal foil, and various plastics, such as polyethylene terephthalate (PET), polyethylene naphthalate (PEN), poly-dimethyl siloxane (PDMS), and polyimides (PI), as substrates.^2−4^ These materials are typically used as passive substrates having no active role in the applications. Moreover, post usage, these plastics can cause serious environmental concerns due to their non-biodegradable nature and thus the resulting accumulation in the environment, also referred to as “white pollution”.^5^ The dependence on the finite and depleting non-renewable petroleum resources for plastic materials is also a major concern. Hence, there is a pressing need for renewably sourced (bio-based) and biodegradable material alternatives, which can match the performance of conventional materials in optoelectronics applications.

A renewable and biodegradable material that is promising in addressing these concerns is cellulose, a linear polymer that is the building block of the structural units of plant fibers. These cellulose fibers can be reduced to the nanoscale through different well-studied chemical, enzymatic, and/or mechanical processes to obtain nanocellulose, a material with fascinating properties.^6,7^ Nanocellulose is a family of a wide range of materials with different nanocellulose grades having varied morphology, functionality, and applications.^8^ There is a widespread appeal of nanocellulose attributable to its salient features, such as mechanical robustness, large specific surface area, high aspect ratio, biocompatibility, and surface accessible hydroxyl groups that can be modified chemically to provide additional functionalities.^9,10^

Two major classes of nanocellulose materials are cellulose nanofibrils (CNFs) and cellulose nanocrystals (CNCs). Typically, CNF grades are composed of highly entangled high aspect ratio nanofibrils which make strong gel networks due to fibril entanglement.^11^ In contrast, CNC grades comprise highly crystalline short rod-like particles.^12^ The exact morphology of both materials depends on the raw material and the used processing conditions. Typically, both CNF and CNC form translucent hydrogels, with CNF suspensions showing a lower gel concentration than CNC suspensions. Nonetheless, both materials exhibit self-assembling propensity and produce dense, transparent standalone films.^13^

The application of CNF films as building blocks and supporting platforms for functional devices in electronics and photonics has been reported frequently during the last decade, as covered in several comprehensive reviews.^14−17^ CNF films exhibit excellent features for optoelectronic devices, such as high mechanical strength,^1^ high light transmittance,^1,18,19^ low coefficient of thermal expansion,^20^ dimensional and chemical stability,^21,22^ and a few other interesting optical properties.^23,24^ Moreover, CNF films can act as superb matrix materials for encapsulating a variety of fillers for preparing functional composites.^25−28^ On the other hand, CNC films are mechanically brittle and exhibit high transmittance and low haze but have similar thermal and chemical properties as CNF films.^7^ A salient feature of CNCs is their ability to produce chiral nematic, iridescent films, which has led to a comprehensive exploration of their applications in photonics.^29,30^ Besides, both CNF and CNC films are biodegradable and eco-friendly and can be easily disposed of in the same manner as traditional paper.^31^

Thermochromism is a phenomenon where a material changes its color as a response to temperature variation in a specific temperature range.^32^ The thermochromic effect can be reversible or irreversible, and the color change can be from one color to another color or from colorless to colored and vice versa. Thermochromic materials have been of interest to the scientific community for the past several decades due to their extensive potential applications in temperature indicators, protective equipment, design accessories, wearable displays, smart windows, sensors, drug delivery, aerospace, military, anti-counterfeiting technologies, construction, textiles, and printing technologies.^33−36^ Thermochromic materials can also potentially be useful for imparting special functionality to nanocellulose films, enabling their use in a wide range of novel applications. Surprisingly, this application area has rarely been explored in the literature. Recently, CNCs were incorporated into ethylene vinyl alcohol copolymer, and photochromic and thermochromic nanocomposite films with repeatable color change properties were prepared.^2^

Optical modulators are devices used, for example, to manipulate transmitted light intensity, phase, and polarization. They find applications in controlling light transmission, for instance, in telecommunication, sensing, and smart-environment applications.^37^ Optical modulation can be achieved using various techniques such as heating and electric or entirely optically (termed as all-optical modulation). For instance, Cocorullo and Rendina demonstrated thermo-optical modulation in a silicon etalon where light transmission was altered in a silicon Fabry-Perot etalon at 1.5 μm by heating.^38^ Electro-optic modulation was presented by Xu et al.^39^ where a 1.5 μm range signal was modulated electrically with 1.5 Gbit/s frequency varying refractive index in a 12 μm diameter silicon ring resonator. Almeida et al.^40^ demonstrated refractive index change-based all-optical modulation where the refractive index in a silicon ring resonator was changed using a picosecond laser with a 78 MHz repetition rate to obtain maximum signal modulation of 94%. Benítez et al.^41^ used a 532 nm laser and Joule heating to tune light transmission through a graphene-based device where they achieved kHz-range modulation with the laser heating and Hz-range modulation with the electrical heating.

**Figure 1:**
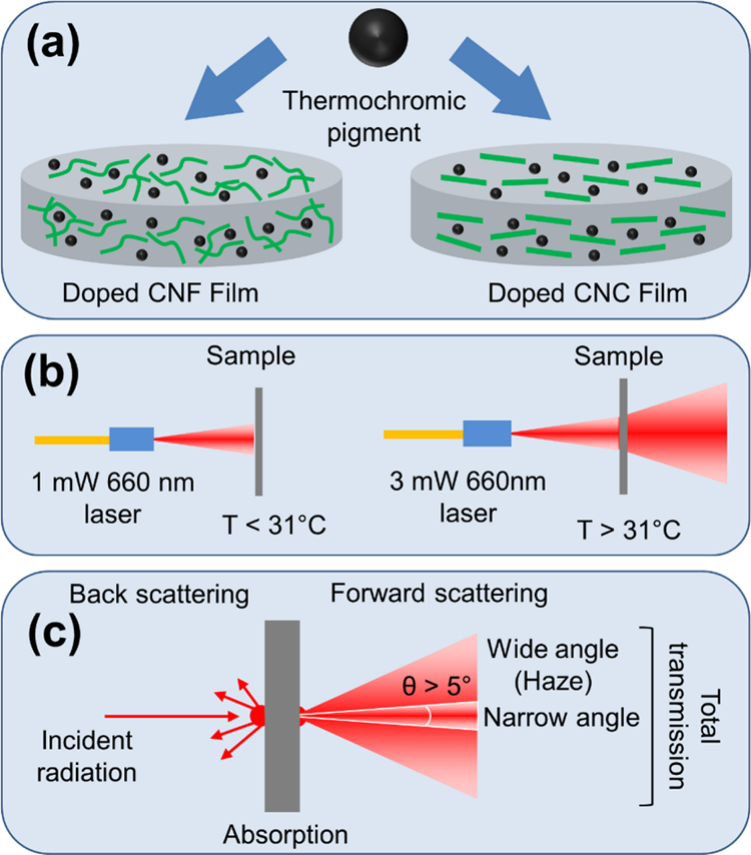
Schematic depiction of the presented work. (a) Illustration of the composite nanocellulose films with thermochromic pigment as the filler for CNF and CNC matrices. The CNF matrix is composed of highly entangled nanofibrils, whereas the CNC matrix is composed of short rod-like crystalline particles. (b) Depiction of the optical characterization of the sample films using a laser light source. Laser intensity was varied to generate a thermochromic transition in the films, rendering them transparent when locally heated above 31 °C via a laser. The shift in optical properties upon the thermochromic transition was studied in terms of transmission, forward and backward scattering, haze, and attenuation. (c) Description of the visible laser light interaction with the sample film and optical parameters measured in the current study.

To bring together all the aspects described above, in this work, we present a concept to produce an all-optical light modulation device prepared using nanocellulose hybrid films. An additional objective was to explore the impact of nanocellulose morphology on the thermally induced optical response of the films. Hybrid films were fabricated using CNF and CNC as matrix materials and installing a thermochromic pigment to these matrices. The pigment imparted thermoresponsive functionality to the nanocellulose films, and thus, the optical properties of the films could be tuned remotely using heat and light. Lastly, the tunability of optical properties was harnessed to demonstrate an all-optical light modulator made from the films for potential applications in sensing systems. A schematic description of the work is shown in Figure 1. To the best of our knowledge, there is no previous work in the literature where such an application for nanocellulose films has been reported. Hence, this work could be at the frontier for further development of nanocellulose films in temperature- or light-sensing applications.

## Experimental Section

### Materials

The CNF suspension was produced via mechanical homogenization of native bleached softwood kraft pulp in a grinder (Supermasscolloider MKZA10-15J, Masuko Sangyo Co., Japan) at 1500 RPM. Subsequent to the homogenization step, the fiber suspension was fed into a microfluidizer (M-7115-30, Microfluidics, USA). A total of seven passes through the microfluidizer were made at 1800 bar pressure. The first pass utilized chambers with diameters of 500 and 200 μm, and the following six passes were through 500 and 100 μm chambers. A white-colored viscous gel was obtained after the treatment with a final solid content of 1.7%.

CNCs were procured from CelluForce, Canada (CelluForce NCC) as a spray-dried powder. The nominal sulfate content of the CNC was 0.86–0.89% and the crystallinity was 88% according to the manufacturer. Further investigation of CelluForce NCC material has been reported elsewhere.^42^ CNC films were found to be extremely brittle and difficult to handle. This issue has been reported in several works previously, and the addition of plasticizers has been shown to decrease film brittleness. Koppolu et al.^43^ have suggested the use of sorbitol as a plasticizer for CNC films. Therefore, 30% (on dry weight) of D-sorbitol (Sigma-Aldrich GmbH, Germany) was added to the CNC suspension to improve film handling.

Leuco dye-based thermochromic pigment slurry was provided by LCR Hallcrest Ltd., United Kingdom (Trade name: ChromaZone Black) as an aqueous suspension. The pigments were black colored, and black to colorless transition occurred at a nominal temperature of 31 °C. The use of the same pigment and its brief description has been reported elsewhere.^35^ Black pigments were chosen to be incorporated in transparent cellulosic films to demonstrate film applications in light blocking and to enable high contrast during experiments. Four different pigment addition levels, viz., 5, 10, 20, and 30% (on dry cellulose mass), were used in the study to determine the effect of pigment incorporation into the cellulose hybrid films.

## Methods

### Fabrication of Thermochromic Hybrid Films

Thermoresponsive cellulose composite films were prepared via solvent casting. The target basis weight of the films was 50 g/m^2^ to enable easy handling. Measured amounts of water-based suspensions were poured into Petri-dishes of known size and dried at 23 °C and 50% relative humidity to obtain standalone films. The casting consistencies of the CNF and CNC suspensions were 1.2 and 3%, respectively. The thermochromic pigment slurry was mixed with the nanocellulose suspensions for 30 min to ensure homogeneity in the casting mixture. Prior to casting, all suspensions were deaerated using an asymmetric vacuum-aided centrifuge (SpeedMixer DAC 600, Synergy Devices Ltd., UK).

### Physical Characterization of Films

Film thickness was measured using an L&W Micrometer 051 (Lorentzen & Wettre AB) following the TAPPI T411 standard.^44^ Basis weight of the films was measured by weighing a film sample of known size. Film density was calculated from the thickness and basis weight values. Scanning electron microscopy (SEM) images of the dried film samples were taken with a field emission scanning electron microscope (Carl Zeiss Merlin) using a secondary electron detector at an acceleration voltage of 3.0 kV and a probe current of 60 pA. Sample surfaces were sputter-coated with a thin gold-platinum layer before imaging, and all images were taken at 2048 × 1536 pixel resolution.

### Forward Scattering Angle with a Red Diode Laser

Forward scattering angle was measured with a red 660 nm diode laser (LPS-660-FC, Thorlabs Inc., USA). The 4.2 mW laser light was first collimated with a single-mode fiber (SM600, Thorlabs) and a fiber lens (F230FC-1550, Thorlabs) before it passed through the sample film onto a screen. The scattered beam width was measured using the 1/e^2^ method for a Gaussian beam.^45^ Measurement data have been reported as normalized intensity as a function of scattering angle from the laser beam axis. Signal noise was filtered using the adjacent averaging method with a window size of 30 points using OriginPro 2019 software. The measurement data were also fitted to the Gaussian function using OriginPro 2019 software, and the full width at half-maximum (FWHM) values were derived from the fitted curves. The fitted plots and the FWHM values can be found in the Supporting Information.

### Scattering, Transmission, and Attenuation Measurements with an Integrating Sphere

**Figure 2:**
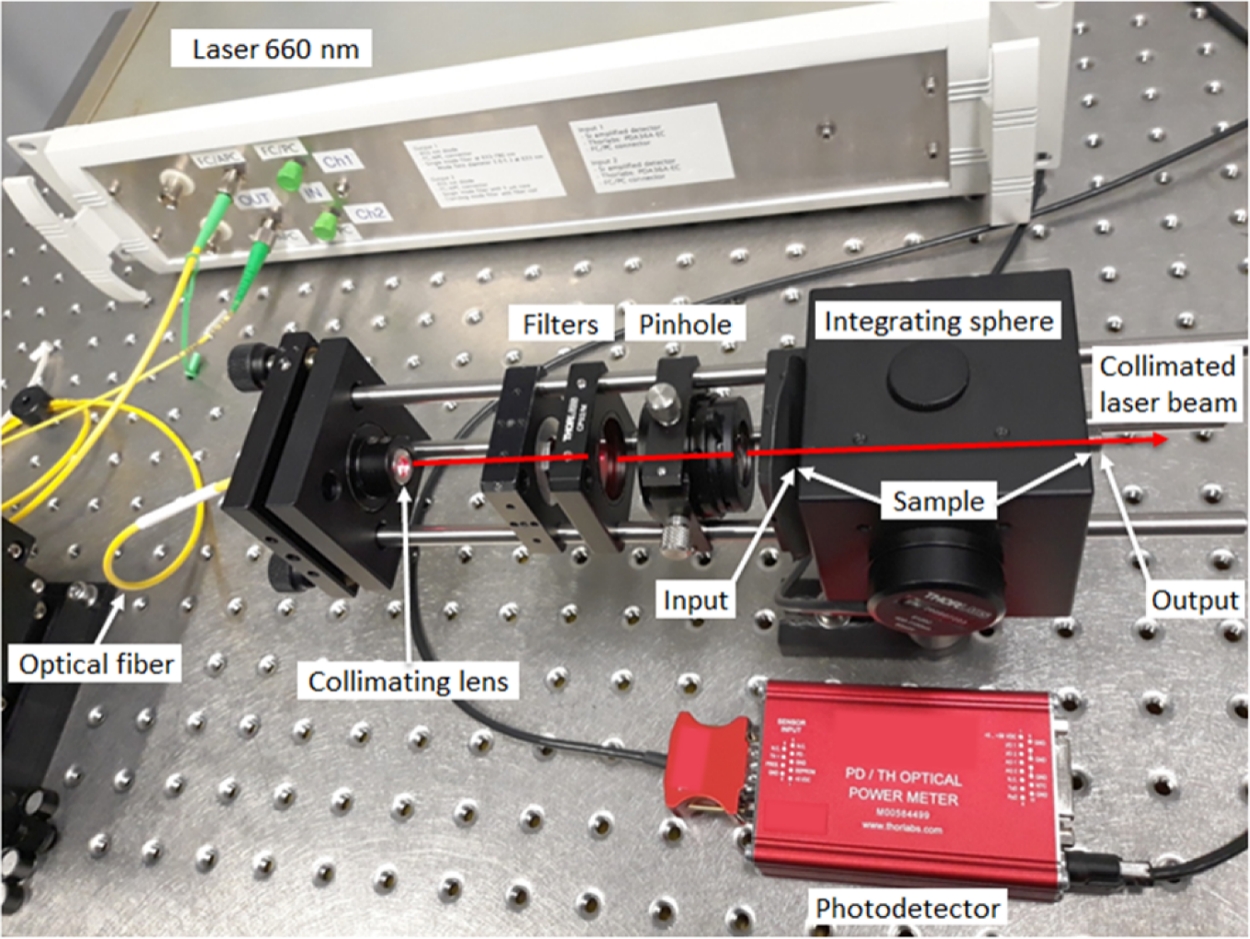
Integrating sphere for light-scattering, transmission, and attenuation measurements.

Light-scattering measurements were performed using an integrating sphere (IS200-4, Thorlabs Inc.) with a polytetrafluoroethylene (PTFE) inner coating giving a nominal 99% spectral reflectance for 350–1500 nm wavelength range. Scattering, transmission, and attenuation were all measured with the same system. The measurement setup is shown in Figure 2. Laser light (660 nm) was first collimated with a single-mode fiber (SM600, Thorlabs) and a fiber lens (F230FC-1550, Thorlabs) and then passed through optical filters, a pinhole, and finally, the integrating sphere. The pinhole was used for laser beam alignment. Different optical filters were used to tune the incident laser beam intensity ranging from 0.3 to 4.2 mW (S10-660-F-S207 from Corion Inc., HQ505LP from Chroma Technologies Inc., and 640LP from Omega Optical Inc.). Light intensity was measured with a Thorlabs PM101 power meter interface and an S120C Photodiode sensor. Measured signals from the power meter interface were collected with LabView software.

Transmission and forward scattering were measured through the film sample that was placed between a PTFE-coated 2.5 mm diameter connector and the input port of the integrating sphere. The output port of the integrating sphere was blocked during transmission measurements and fitted with a PTFE-coated 3.2 mm diameter connector in forward scattering measurements. The diameter of the output port (3.2 mm) defines the proportion of the forward scattering that can be termed as Transmission Haze. During backscattering measurements, the film sample was placed between the output port and the 2.5 mm connector, while the 3.2 mm connector was on the input port.

### Halogen Lamp and Optical Spectrum Analyzer for Transmission Spectrum Measurements

Light absorption spectra of the film samples were measured with a setup involving a halogen lamp (HK-2000-HP, Ocean Optics Inc.) and an optical spectrum analyzer (OSA) (AQ-6315A, Ando Electric Ltd.). A multimode optical fiber (Thorlabs) (105/125 mm core/cladding diameters) from the halogen lamp was coupled to a fiber collimator (F230FC-1550, Thorlabs). Transmitted light was collected with the same kind of fiber collimator and a multimode optical fiber (Thorlabs) 200/220 mm (core/cladding) to the OSA. Light absorption spectra of the film samples were measured in the 350–1750 nm wavelength range with 10 nm resolution.

### Infrared Light Modulation with a Visible Red Diode Laser

**Figure 3:**
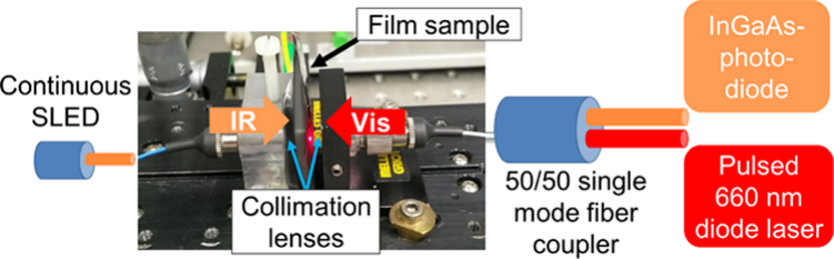
Setup for measuring continuous IR light modulation with a pulsed visible diode laser.

Continuous infrared (IR) light from a superluminescent light-emitting diode (SLED) (1310 nm, 17.8 mW, 85 nm bandwidth) was modulated with a 0.3 Hz pulsed visible diode laser (660 nm, 6.3 mW, LPS-660-FC, Thorlabs). IR and visible light were coupled to the opposite direction in an air cavity that was made with fiber collimators (F230FC-1550, Thorlabs) in a setup shown in Figure 3. The air cavity was approximately 1 mm long. A single-mode fiber coupler (50/50%, PN1550R5F1, Thorlabs) coupled modulated IR light to an InGaAs photodiode (S155C, Thorlabs) equipped with a PM101 Power meter interface. The coupler also coupled the diode laser to the air cavity. The used photodiode did not detect visible range laser. Signal data was collected from the power meter interface using LabView software.

## Results and Discussion

### Thermochromic Functional Films

**Figure 4:**
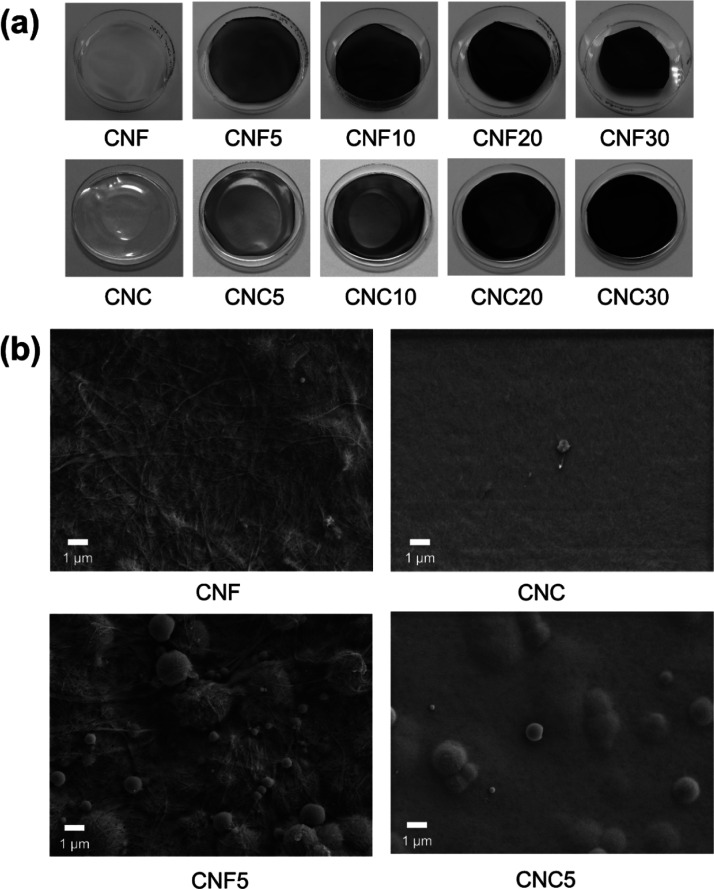
(a) Digital photographs of all film samples at 23 °C and (b) SEM micrographs of pure nanocellulose and doped films (5% pigment loading) at 5000× magnification. The SEM image of pure CNC film in (b) focuses on an artifact.

Two different types of nanocellulose hybrid films were prepared in this present work using CNF and CNC as matrix materials, in order to study the effect of morphology on the formation of hybrid film structures. Both nanocellulose materials were doped with a leuco dye-based thermochromic pigment at four different addition levels, viz., 5, 10, 20, and 30% based on dry cellulose mass. Since the pigment was black at room temperature, the color of the films was observed to darken with pigment addition (Figure 4a). Despite pigment addition, the film formation was found to be excellent in all samples; however, slight wrinkling could be observed in CNF films at filler levels of 20 and 30%. All film samples were found to have uniform thickness with low deviation, adequate for both inter- and intra-sample comparison. The sample nomenclature and selected properties have been enlisted in Table 1. It must be noted that in further discussion, the terms “CNF films” and “CNC films” correspondingly represent all CNF- and CNC-based samples collectively, unless a specific sample code is mentioned.

**Table 1:** Description of the Prepared Films and Selected Film Properties

matrix material	sample code	pigment addition (%)	film thickness (μm)	film density (g/cm^3^)
CNF	CNF	0	36.0 ± 3.1	1.39 ± 0.10
	CNF5	5	35.5 ± 2.2	1.36 ± 0.09
	CNF10	10	41.7 ± 5.5	1.27 ± 0.16
	CNF20	20	38.0 ± 0.8	1.36 ± 0.06
	CNF30	30	37.7 ± 1.9	1.38 ± 0.06
CNC	CNC	0	34.6 ± 2.7	1.47 ± 0.12
	CNC5	5	34.4 ± 3.9	1.50 ± 0.17
	CNC10	10	36.0 ± 3.7	1.50 ± 0.16
	CNC20	20	33.4 ± 2.7	1.45 ± 0.12
	CNC30	30	37.6 ± 5.4	1.42 ± 0.21

CNF and CNC have vastly different morphologies. CNF grades are composed of highly entangled high aspect ratio fibrils,^11^ whereas CNC grades have rod-like crystals with a relatively low aspect ratio.^46^ Their morphological dissimilarity is reflected in the films formed via self-assembly by CNF and CNC grades, which exhibit distinct properties such as density, strength, flexibility, transparency, and haze.^13,47^ In the current work, we visually examined the film formation via SEM imaging of the film surfaces. SEM images revealed that the CNF films possessed a rough, fibrous surface, whereas the CNC film surfaces were rather smooth. For the doped samples, encapsulation of thermochromic particles in the nanocellulosic matrix was also considerably different, where particles were observed to be encapsulated in the CNF matrix in a “spider-web”-like manner (Figure 4b). In the case of CNC-based films, the particles appeared to be uniformly covered with CNC.

### Optical Properties and Thermal Response

In the present work, the main emphasis was paid on investigating the optical properties of the prepared functional films and studying their thermal response. Different optical applications of nanocellulose films demand target-specific properties. For instance, high optical transmission and high wide-angle forward scattering (high haze) are demanded in solar cell substrate applications,^48^ whereas high transmission and low haze are required for applications such as packaging and displays.^49^

### Forward Scattering Behavior

**Figure 5:**
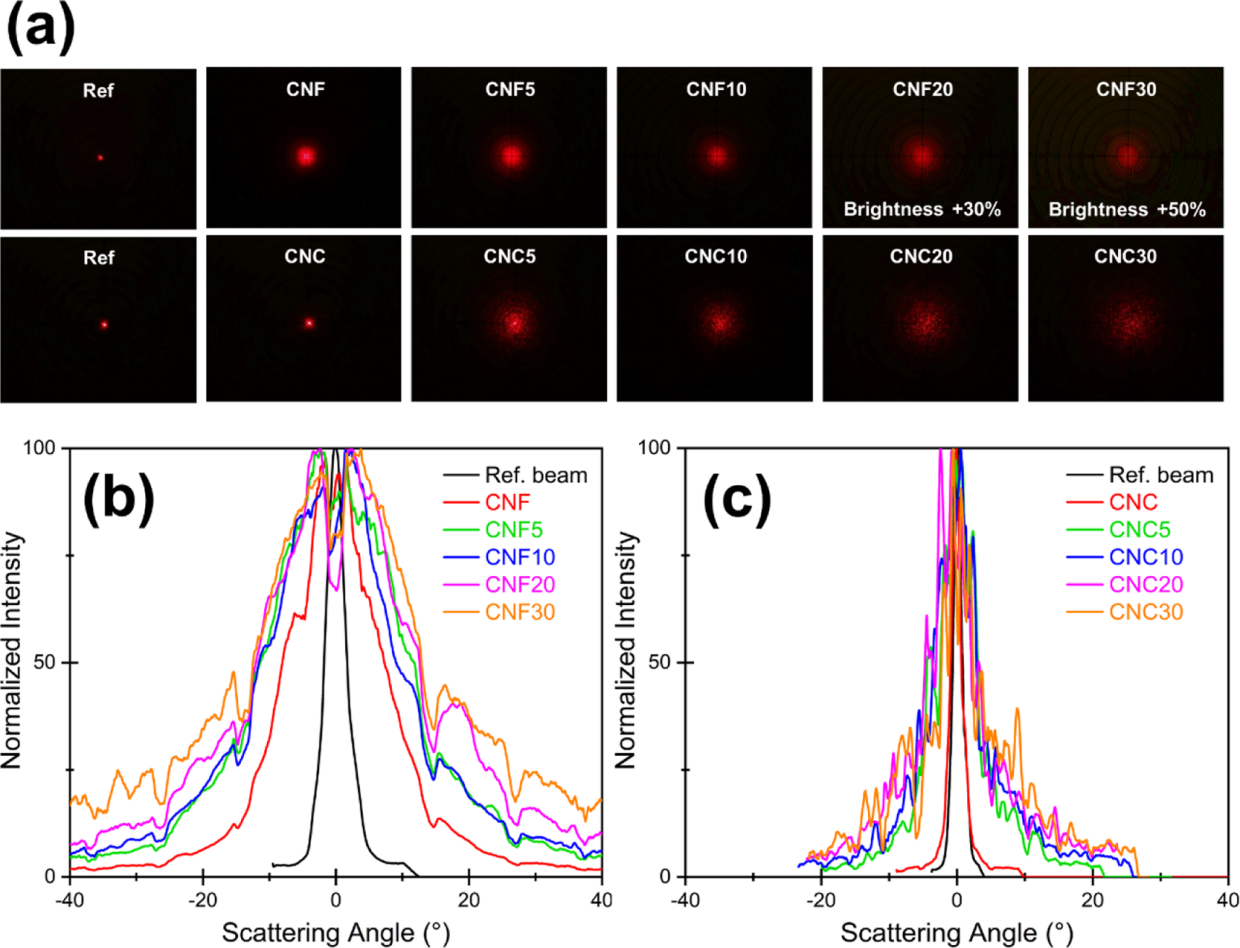
(a) Forward scattering from the film samples imaged at a distance of 40 mm for CNF films and at 113 mm for CNC films using an incident 660 nm laser beam. Brightness of CNF20 and CNF30 samples has been increased by 30 and 50%, respectively, to enhance the visibility. (b,c) Normalized intensity distribution measured at different scattering angles from the laser beam axis for the CNF and CNC samples, respectively.

The forward scattering angle was determined using a collimated laser beam as a reference. Light scattered after passing through the films was collected on a screen and intensity distribution was measured. Digital photographs from the screen for different samples, when illuminated with the laser beam, are shown in Figure 5a. The increment in beam width upon scattering is already noticeable from the images. Nonetheless, the scattering angles were calculated from the intensity distribution and have been illustrated in Figure 5b,c for CNF and CNC films, respectively. It must be noted that the images in Figure 5a, along with scattering, also include the laser interference that comes from the nanocellulose material itself. This adds noise to the scattering angle distributions and limits the measurement resolution to degree level.

As shown in Figure 5, it was observed that CNF films exhibited much higher forward scattering than CNC and scattered the laser beam widely. Thus, in order to collect all scattered light, the screen had to be placed at a lower distance for the CNF samples (40 mm) than for the CNC samples (113 mm). Even in that case, a clear difference between the CNF and CNC films was observed. The scattering angle for the CNF films was measured to be 27–68°, whereas for CNC films, it was much lower, ranging from 3 to 23°, thus ascertaining a higher transmission haze in CNF films, as reported previously in the literature.^50^ A similar trend was observed from the FWHM values calculated from the Gaussian fits for the same data where the FWHM values for CNF films ranged from 16 to 30° and only from 4 to 10° for the CNC films (shown in the Supporting Information). This result can be correlated to the physical structure of the films where light-scattering interfaces can be present either in the film bulk or on the surface.^51^ CNC films were measured to have higher densities than CNF films (Table 1), which translates into a lower number of scattering interfaces in the film bulk, thus producing a lower haze. Another reason behind a higher haze in the CNF films could be the presence of fibril aggregates in the CNF films, which accentuates light scattering.^52^ The effect of surface roughness and porosity of the CNC films on their light-scattering behavior has been previously reported by Nogi et al.^18^ Another observation from this experiment was that as the pigment loading was increased, the scattering angle also increased (wider intensity distribution curve). This is probably a result of the addition of microsized pigments (*D*_97_ < 6 μm) which created more scattering interfaces in the film, hence enhancing the scattering effect.

### Scattering, Transmission, and Attenuation via an Integrating Sphere

Further investigation focused on studying the thermal response of the film in terms of absolute forward and back scattering, transmission, and attenuation. The measurements were performed on an integrating sphere using four different 660 nm laser intensities (0.3, 1.9, 3.4, and 4.2 mW). The two selected higher laser powers of 3.4 and 4.2 mW were found adequate to heat the film locally to allow reversible thermochromic transition and thus cause a substantial shift in the optical properties. The heating effect of different laser powers was ascertained using a thermal imager before measurements.

**Figure 6:**
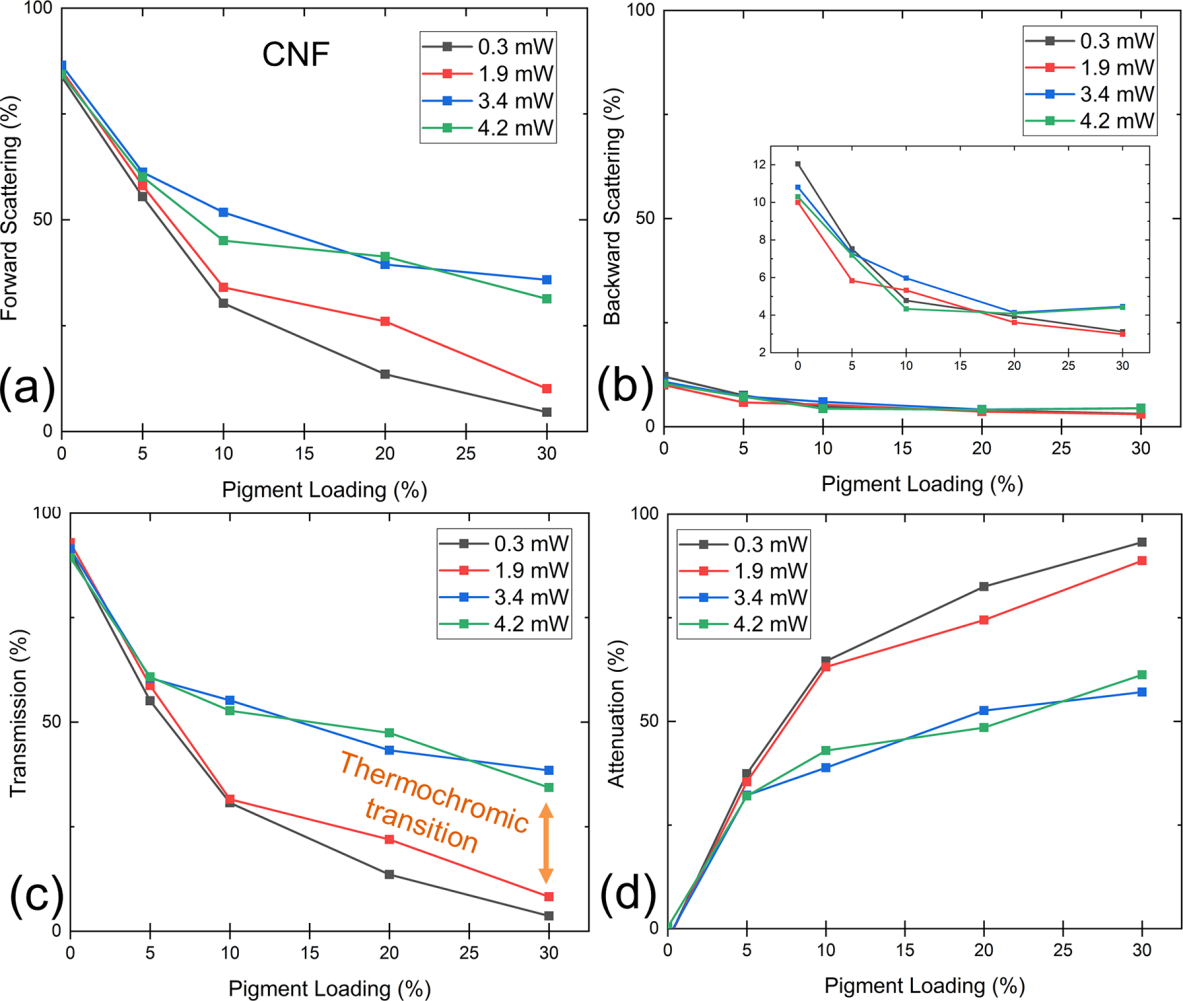
(a) Forward scattering (transmission haze), (b) backward scattering, (c) transmission, and (d) attenuation for CNF films as a function of thermochromic pigment loading level. The inset in (b) shows the same curves when the vertical axis is magnified. Thermochromic transition, producing a significant difference in transmission, is annotated in subfigure (c).

As described above, pure CNF films exhibit a high light-scattering tendency. Almost all transmitted light was found to be scattered as haze (Figure 6a), and the attenuation of pure CNF film was almost zero (Figure 6d). For the undoped CNF films, the transmission was greater than 90%. In the literature, for 660 nm wavelength irradiated on pure films (25 μm thick) made from a similar CNF grade, Kumar et al.^52^ reported a transmittance of roughly 80%.

The laser light attenuation increased with increasing thermochromic pigment loading. With low laser intensity, which did not cause a thermochromic transition, the attenuation increased to roughly 90% for the CNF30 sample, with the film hardly transmitting any light. However, when illuminated with higher intensities, the films heated locally, thus causing a leuco transition and a resultant drop in attenuation (ca. 60% for the CNF30 sample at 3.4 mW). The same effect can also be observed in the transmission results (Figure 6c) where the occurrence of the thermochromic transition has been labeled in the figure. The transmission increased from 4 to 38% for the CNF30 sample when laser power was changed from 0.3 mW to 3.4 mW. Haze was found to follow a similar trend with respect to pigment loading as transmission. We hypothesize that the decrease in transmission and haze originated primarily from the attenuation caused by the black pigment. Backward scattering from CNF films was low but decreased from 10–12% to below 5% as the pigment loading increased from 0% to 30%.

Further, we examined the thermal response of the CNC films in a similar manner. As shown previously in Figure 5, CNC films have much lower scattering angles as compared to CNF films. The transmission haze for pure CNC films varied from 10 to 30%, whereas it was approximately 90% for the pure CNF sample. The transmission for both types of films was similar, ranging from 88 to 92%. These values are higher than those recently reported by Wang et al.^47^ who reported transmittance of about 70% for 20 μm thick CNC films (at 660 nm). This difference might have arisen due to morphological dissimilarity between the two CNC grades.

**Figure 7:**
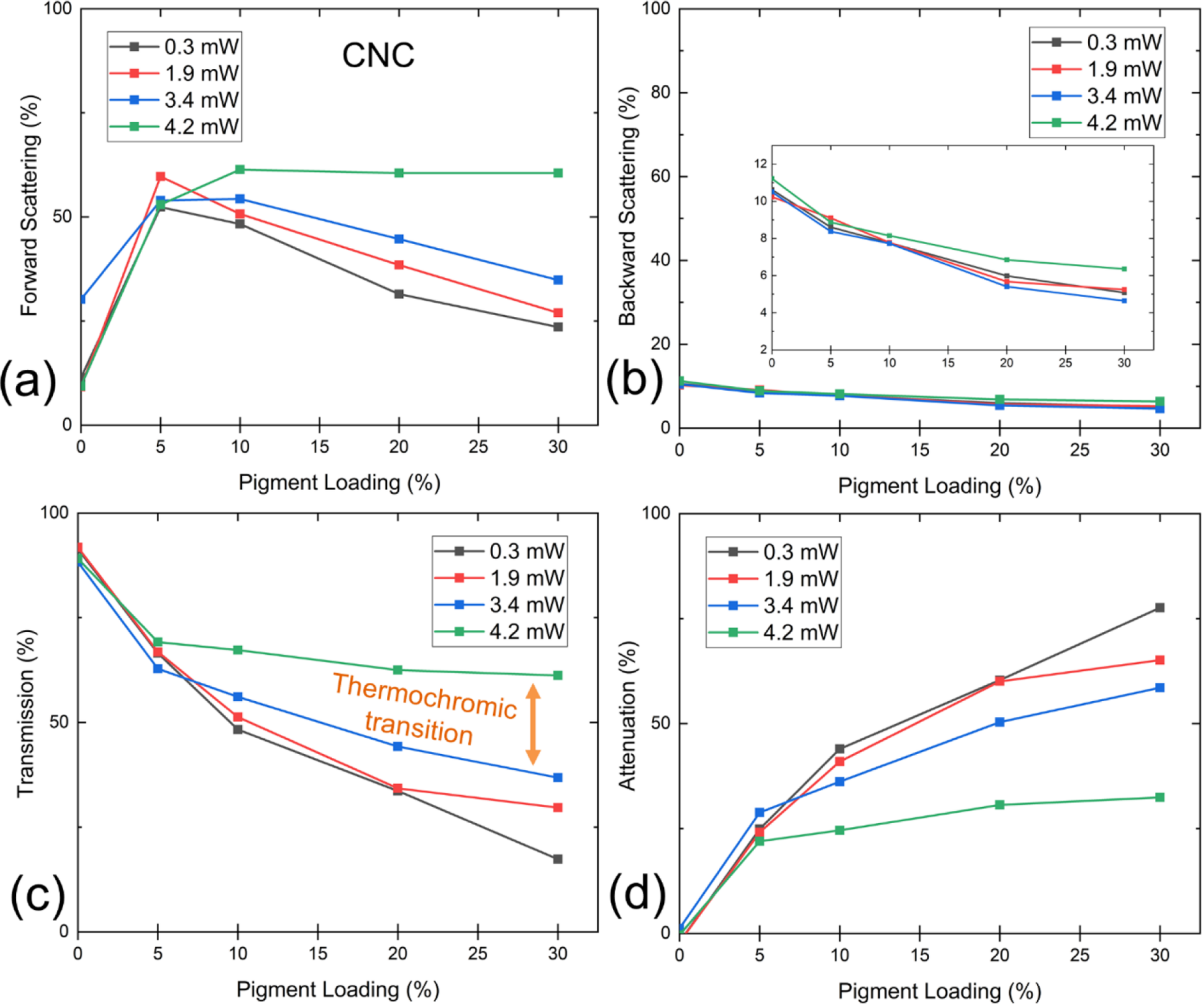
(a) Forward scattering (transmission haze), (b) backward scattering, (c) transmission, and (d) attenuation for CNC films as a function of thermochromic pigment loading level. The inset in (b) shows the same curves when the vertical axis is magnified.

The optical properties of the doped CNC film samples were rather peculiar. The transmission haze increased to 50–60% already at 5% pigment loading, as compared to 8–30% for the pure film (Figure 7a). However, with further increase in the pigment loading, an inflection in the curve was observed as the haze dropped to 23–40% in the absence of thermochromic transition (while remaining at 58 ± 3% with 4.2 mW laser power). This behavior is quite different from CNF films, where the haze was found to be a globally falling function. This trend can be attributed to the increase in attenuation due to the presence of a high amount of pigment in the film. As shown in Figure 7d, the attenuation increased from almost zero to over 70% with 0.3 mW laser intensity, while pigment concentration changed from 0 to 30%. Nonetheless, beyond thermochromic transition, the attenuation decreased greatly to 27 ± 4%.

The effect of attenuation can also be observed in the absolute transmission data (Figure 7c) where the transmission was measured to be 90 ± 1% for pure film and it decreased to 17 ± 1% for the CNC30 sample (at 0.3 mW power). It is interesting to note that even though high pigment loading increases attenuation, it also accentuates the magnitude of the thermochromic effect. For instance, upon thermochromic transition, the transmission increased by 44% points for the CNC30 sample, meanwhile increasing by ∼29, ∼19%, and only ∼3% points for CNC20, CNC10, and CNC5 samples, respectively. The backward scattering of CNC films was low, and the behavior was alike CNF films. The backward scattering values ranged from 10 to 12% for pure CNC films and decreased to 4–6% level as a function of pigment loading.

### Light Transmission Spectra

**Figure 8:**
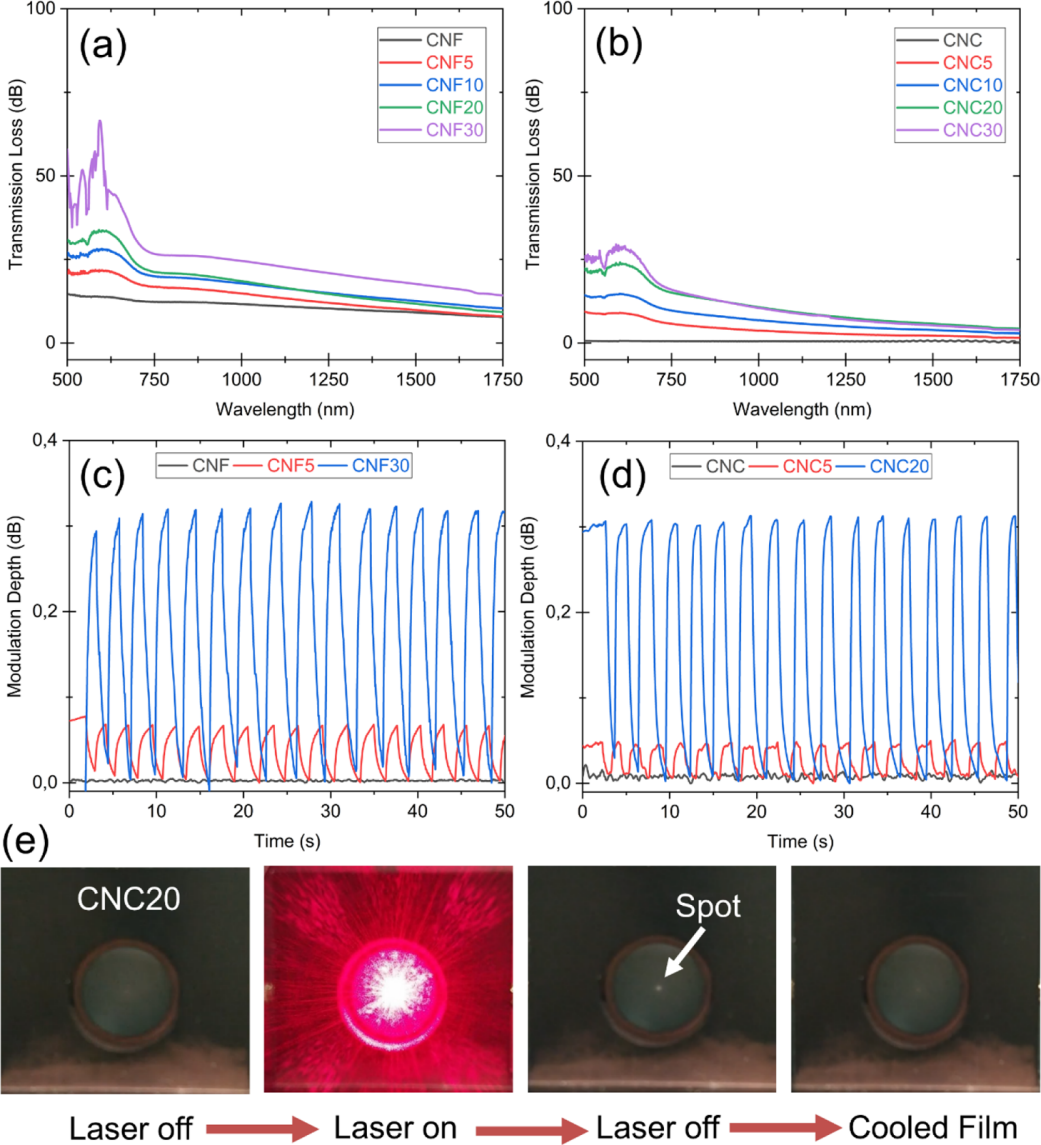
Transmission loss in air cavity for (a) CNF- and (b) CNC-based samples under illumination by a halogen lamp. (c,d) Continuous IR light modulation depths exhibited by CNF- and CNC-based samples, respectively, under a 6.2 mW pulsed visible diode laser. The modulation depth has been shown for the control samples, and the samples showing the lowest and highest performance. (e) Images of the CNC20 sample during the modulation tests.

Absorption spectra of thermochromic films were measured using an OSA by illuminating the films with a halogen lamp. Transmission loss through the films was calculated and is illustrated in Figure 8a,b. Both undoped CNF and CNC films did not show any absorption band in the visible or IR range. The presence of thermochromic pigment produced an absorption maximum near 600 nm, and the transmission loss amplified as the pigment loading increased. Strong absorption of the doped films in this wavelength range also explains quick temperature increase even with low laser intensities at 660 nm.

### Demonstration of All-Optical Modulation

After investigating the optical properties and the thermal response of the prepared films, we studied the thermochromic functional films in a totally novel application as an all-optical modulator. Previously, a graphene-based thin-film device^41^ has been demonstrated as a thermoresponsive all-optical modulator; however, the use of nanocellulose films in this domain is non-existent.

Continuous IR light modulation using a visible red diode laser was tested using both CNF and CNC films. A custom-built setup was used for modulation studies (see Experimental Section). IR and visible light were collimated in opposite directions in an air cavity, and the film samples were put to this air cavity such that IR and visible light were coupled to the same spot with the same optical path length. The diode laser intensity was measured to be 6.3 mW in the sample position. Using a thermal imager, it was ascertained that the laser power was sufficient to raise the sample temperature above 31 °C and cause a local thermochromic transition at the measurement spot. The visible diode laser was pulsed at 0.3 Hz frequency, and the IR light transmission was measured. Low pulsing frequency was chosen to enable cooling of the film below 31 °C between laser pulses. Figure 8e visually depicts the spot heating and cooling cycle of the CNC20 sample with laser as an example where the creation of a reversible transparent spot is evident.

Figure 8c,d shows the modulation depth exhibited by the CNF and CNC films, respectively. Here, the modulation depth denotes the magnitude of IR light (as compared to the reference level) that passed through the sample each time it was illuminated by the laser light. As expected, pure film samples did not exhibit any modulation in the absence of thermochromic functionality. However, weak modulation (<0.1 dB) was already observed in CNF5 and CNC5 samples. The modulation depth for CNF films increased as a function of pigment loading; meanwhile, CNC films showed a maximum at 20% pigment loading. The best performing samples were CNF30 and CNC20, both showing a similar 0.3 dB modulation depth. Moreover, the modulation cycles were quite repeatable for both types of films with the CNC20 sample having slightly better cycle uniformity.

Modulation depth could be controlled by varying the laser pulsing frequency. Higher depth can be achieved by using a lower frequency as it increases the spot heating duration. A modulation depth of 2 dB was achieved by heating samples for 120 s. However, this frequency is quite slow for many sensing applications; hence, a lower frequency (0.3 Hz) was used for detailed study, as shown in Figure 8. An exciting finding of this study was that a reasonable modulation depth of 0.3 dB could be achieved when the complete switching cycle was 3.3 s long. These results indicate that the demonstrated functional nanocellulose films can be used as a modulation device for thermal sensing applications that require a similar response time.

## Conclusions

In this work, we presented nanocellulose composite films with thermoresponsive optical functionality. The films were prepared using CNF and CNC as matrix materials and a leuco dye-based thermochromic pigment as a filler. Due to the morphological dissimilarity between the materials, films prepared from CNF and CNC exhibited a difference in their structures and thus exhibited distinct optical properties such as transparency and light scattering.

Pure CNF and CNC films had similar transparency (88–92%) but significantly different transmission haze (∼90% as compared to ∼30%). The conical scattering angle was measured up to 70° for CNF films, whereas the value was below 25° for CNC films. The addition of black thermochromic pigment to the nanocellulose matrices impacted the optical properties in terms of reduced transparency and higher scattering. Increasing pigment loading was found to increase light attenuation through the film while also intensifying the magnitude of the thermochromic effect. For CNF films with 30% pigment loading, light transmission increased 9-fold upon thermochromic transition; meanwhile, the increase was only 1.1 times at 5% pigment loading. Similarly, for CNC films, the transmission increased 3.5 times and 1.2 times at 30 and 5% pigment loading levels, respectively. The significant shift in the optical properties of the films in response to heating was harnessed in a light switch application.

Continuous IR light modulation using a visible diode laser was demonstrated using both CNF and CNC films. Pure nanocellulose films did not exhibit any modulation, while weak modulation (<0.1 dB) was already observed at 5% pigment loading. The best performing samples, CNF30 and CNC20, exhibited a maximum repeating modulation depth of 0.3 dB when pulsed with laser light at 0.3 Hz frequency.

The all-optical light modulator demonstrated in the present work could be utilized in various thermally stimulated sensing systems such as indoor temperature monitoring, water heating, energy-saving switches, and anti-counterfeiting. Nonetheless, thermoresponsive functional nanocellulose films can also find other suitable applications apart from modulation. In this work, only one type of thermochromic pigment was studied; however, these pigments can be tuned to work in a wide temperature range, which broadens the applicability of presented composite films. Thus, further studies could focus on using different kinds of thermochromic materials and examining their surface interaction with nanocellulose materials. Future work on the topic could also include studying the thermal response of the films made from other cellulosic materials, such as cellulose derivatives, which also produce strong and optically transparent films of biobased nature. Another aspect for further exploration could be discovering more and novel applications of such functional films.

## Supporting Information Available

Additional SEM images, scattering angle plots, and modulation results (PDF)
Thermoresponsive nature of the CNC film sample (MP4)

## References

[ref1] Zhu H.; Fang Z.; Preston C.; Li Y.; Hu L. Transparent Paper: Fabrications, Properties, and Device Applications. Energy Environ. Sci. 2014, 7, 269–287. Royal Society of Chemistry December 10.1039/c3ee43024c.

[ref2] Li M.; Zhao X.; Li Y.; Wang W.; Zhong W.; Luo M.; Lu Y.; Liu K.; Liu Q.; Wang Y.; Wang D. Synergistic Improvement for Mechanical, Thermal and Optical Properties of PVA-Co-PE Nanofiber/Epoxy Composites with Cellulose Nanocrystals. Compos. Sci. Technol. 2020, 188, 107990 10.1016/j.compscitech.2020.107990.

[ref3] Gao L.; Chao L.; Hou M.; Liang J.; Chen Y.; Yu H.-D.; Huang W. Flexible, Transparent Nanocellulose Paper-Based Perovskite Solar Cells. npj Flexible Electron. 2019, 3, 4 10.1038/s41528-019-0048-2.

[ref4] Zhu H.; Shen Y.; Li Y.; Tang J. Recent Advances in Flexible and Wearable Organic Optoelectronic Devices. J. Semicond. 2018, 39, 011011–011022. 10.1088/1674-4926/39/1/011011.

[ref5] Hameed M.; Bhat R. A.; Singh D. V.; Mehmood M. A. White Pollution: A Hazard to Environment and Sustainable Approach to Its Management Microbiota and Biofertilizers: A Sustainable Continuum for Plant and Soil Health″ View Project Waste Management View Project, igi-global.com, 2020.

[ref6] Isogai A. Wood Nanocelluloses: Fundamentals and Applications as New Bio-Based Nanomaterials. J. Wood Sci. 2013, 59, 449–459. 10.1007/s10086-013-1365-z.

[ref7] Abitbol T.; Rivkin A.; Cao Y.; Nevo Y.; Abraham E.; Ben-Shalom T.; Lapidot S.; Shoseyov O. Nanocellulose, a Tiny Fiber with Huge Applications Current Opinion in Biotechnology; Elsevier Current Trends, 2016; Vol. 39, pp 76–88. 26930621 10.1016/j.copbio.2016.01.002

[ref8] Dufresne A. Nanocellulose Processing Properties and Potential Applications. Curr. For. Rep. 2019, 5, 76–89. 10.1007/s40725-019-00088-1.

[ref9] Moon R. J.; Schueneman G. T.; Simonsen J. Overview of Cellulose Nanomaterials, Their Capabilities and Applications. JOM 2016, 68, 2383–2394. 10.1007/s11837-016-2018-7.

[ref10] Hubbe M. A.; Ferrer A.; Tyagi P.; Yin Y.; Salas C.; Pal L.; Rojas O. J. Nanocellulose in Thin Films, Coatings, and Plies for Packaging Applications: A Review. BioResources 2017, 12, 2143 10.15376/biores.12.1.2143-2233.

[ref11] Lavoine N.; Desloges I.; Dufresne A.; Bras J. Microfibrillated Cellulose - Its Barrier Properties and Applications in Cellulosic Materials: A Review. In Carbohydrate Polymers; Elsevier, October 1, 2012; Vol. 90, pp 735–764. 22839998 10.1016/j.carbpol.2012.05.026

[ref12] Habibi Y.; Lucia L. A.; Rojas O. J. Cellulose Nanocrystals: Chemistry, Self-Assembly, and Applications. Chem. Rev. 2010, 110, 3479–3500. 10.1021/cr900339w. 20201500

[ref13] Xu X.; Liu F.; Jiang L.; Zhu J. Y.; Haagenson D.; Wiesenborn D. P. Cellulose Nanocrystals vs. Cellulose Nanofibrils: A Comparative Study on Their Microstructures and Effects as Polymer Reinforcing Agents. ACS Appl. Mater. Interfaces 2013, 5, 2999–3009. 10.1021/am302624t. 23521616

[ref14] Hoeng F.; Denneulin A.; Bras J. Use of nanocellulose in printed electronics: a review. Nanoscale 2016, 8, 13131–13154. Royal Society of Chemistry, July 21 10.1039/c6nr03054h. 27346635

[ref15] Dias O. A. T.; Konar S.; Leão A. L.; Yang W.; Tjong J.; Sain M. Current State of Applications of Nanocellulose in Flexible Energy and Electronic Devices. Front. Chem. 2020, 8, 420 Frontiers Media S.A. May 10.3389/fchem.2020.00420. 32528931 PMC7253724

[ref16] Luo Y.; Zhang J.; Li X.; Liao C.; Li X. The Cellulose Nanofibers for Optoelectronic Conversion and Energy Storage. J. Nanomater. 2014, 2014, 654512 Hindawi Publishing Corporation 10.1155/2014/654512.

[ref17] Li S.; Lee P. S. Development and Applications of Transparent Conductive Nanocellulose Paper. Sci. Technol. Adv. Mater. 2017, 18, 620–633. 10.1080/14686996.2017.1364976. 28970870 PMC5613913

[ref18] Nogi M.; Iwamoto S.; Nakagaito A. N.; Yano H. Optically Transparent Nanofiber Paper. Adv. Mater. 2009, 21, 1595–1598. 10.1002/adma.200803174.

[ref19] Nakagaito A. N.; Nogi M.; Yano H. Displays from Transparent Films of Natural Nanofibers. MRS Bull. 2010, 35, 214–218. 10.1557/mrs2010.654.

[ref20] Fukuzumi H.; Saito T.; Iwata T.; Kumamoto Y.; Isogai A. Transparent and High Gas Barrier Films of Cellulose Nanofibers Prepared by TEMPO-Mediated Oxidation. Biomacromolecules 2009, 10, 162–165. 10.1021/bm801065u. 19055320

[ref21] Fang Z.; Hou G.; Chen C.; Hu L. Nanocellulose-Based Films and Their Emerging Applications. Curr. Opin. Solid State Mater. Sci. 2019, 23, 100764 Elsevier Ltd August 1 10.1016/j.cossms.2019.07.003.

[ref22] Chen C.; Hu L. Nanocellulose toward Advanced Energy Storage Devices: Structure and Electrochemistry. Acc. Chem. Res. 2018, 51, 3154–3165. 10.1021/acs.accounts.8b00391. 30299086

[ref23] Orelma H.; Hokkanen A.; Leppänen I.; Kammiovirta K.; Kapulainen M.; Harlin A. Optical Cellulose Fiber Made from Regenerated Cellulose and Cellulose Acetate for Water Sensor Applications. Cellulose 2020, 27, 1543–1553. 10.1007/s10570-019-02882-3.

[ref24] Simão C. D.; Reparaz J. S.; Wagner M. R.; Graczykowski B.; Kreuzer M.; Ruiz-Blanco Y. B.; García Y.; Malho J.-M.; Goñi A. R.; Ahopelto J.; Sotomayor Torres C. M. Optical and Mechanical Properties of Nanofibrillated Cellulose: Toward a Robust Platform for next-Generation Green Technologies. Carbohydr. Polym. 2015, 126, 40–46. 10.1016/j.carbpol.2015.03.032. 25933520

[ref25] Lee S.-Y.; Mohan D. J.; Kang I.-A.; Doh G.-H.; Lee S.; Han S. O. Nanocellulose Reinforced PVA Composite Films: Effects of Acid Treatment and Filler Loading. Fibers Polym. 2009, 10, 77–82. 10.1007/s12221-009-0077-x.

[ref26] Hu D.; Ma W.; Zhang Z.; Ding Y.; Wu L. Dual Bio-Inspired Design of Highly Thermally Conductive and Superhydrophobic Nanocellulose Composite Films. ACS Appl. Mater. Interfaces 2020, 12, 11115–11125. 10.1021/acsami.0c01425. 32049475

[ref27] Honorato C.; Kumar V.; Liu J.; Koivula H.; Xu C.; Toivakka M. Transparent Nanocellulose-Pigment Composite Films. J. Mater. Sci. 2015, 50, 7343–7352. 10.1007/s10853-015-9291-7.

[ref28] Bardet R.; Belgacem M. N.; Bras J. Different Strategies for Obtaining High Opacity Films of MFC with TiO2 Pigments. Cellulose 2013, 20, 3025–3037. 10.1007/s10570-013-0025-1.

[ref29] Dumanli A. G.; Van Der Kooij H. M.; Kamita G.; Reisner E.; Baumberg J. J.; Steiner U.; Vignolini S. Digital Color in Cellulose Nanocrystal Films. ACS Appl. Mater. Interfaces 2014, 6, 12302–12306. 10.1021/am501995e. 25007291 PMC4251880

[ref30] Hamad W. Y. Photonic and Semiconductor Materials Based on Cellulose Nanocrystals. Adv. Polym. Sci. 2015, 271, 287–328. 10.1007/12_2015_323.

[ref31] Vikman M.; Vartiainen J.; Tsitko I.; Korhonen P. Biodegradability and Compostability of Nanofibrillar Cellulose-Based Products. J. Polym. Environ. 2015, 23, 206–215. 10.1007/s10924-014-0694-3.

[ref32] Abdellaoui H.; Raji M.; Chakchak H.; Qaiss A. e. k.; Bouhfid R. Thermochromic Composite Materials: Synthesis, Properties and Applications. Polymer Nanocomposite-Based Smart Materials; Elsevier, 2020, pp 61–78.

[ref33] Cheng Y.; Zhang X.; Fang C.; Chen J.; Wang Z. Discoloration Mechanism, Structures and Recent Applications of Thermochromic Materials via Different Methods: A Review. J. Mater. Sci. Technol. 2018, 34, 2225–2234. 10.1016/j.jmst.2018.05.016.

[ref34] Fu F.; Hu L. Temperature Sensitive Colour-Changed Composites. In Advanced High Strength Natural Fibre Composites in Construction; Elsevier Inc., 2017, pp 405–423.

[ref35] Perez G.; Allegro V. R.; Corroto M.; Pons A.; Guerrero A. Smart Reversible Thermochromic Mortar for Improvement of Energy Efficiency in Buildings. Constr. Build. Mater. 2018, 186, 884–891. 10.1016/j.conbuildmat.2018.07.246.

[ref36] Abdollahi A.; Roghani-Mamaqani H.; Razavi B.; Salami-Kalajahi M. Photoluminescent and Chromic Nanomaterials for Anticounterfeiting Technologies: Recent Advances and Future Challenges. ACS Nano 2020, 14, 14417–14492. 10.1021/acsnano.0c07289. 33079535

[ref37] Liang X.; Chen M.; Wang Q.; Guo S.; Zhang L.; Yang H. Active and Passive Modulation of Solar Light Transmittance in a Hybrid Thermochromic Soft-Matter System for Energy-Saving Smart Window Applications. J. Mater. Chem. C 2018, 6, 7054–7062. 10.1039/C8TC01274A.

[ref38] Cocorullo G.; Rendina I. Thermo-optical modulation at 1.5μm in silicon etalon. Electron. Lett. 1992, 28, 83–85. 10.1049/el:19920051.

[ref39] Xu Q.; Schmidt B.; Pradhan S.; Lipson M. Micrometre-Scale Silicon Electro-Optic Modulator. Nature 2005, 435, 325–327. 10.1038/nature03569. 15902253

[ref40] Almeida V. R.; Barrios C. A.; Panepucci R. R.; Lipson M. All-Optical Control of Light on a Silicon Chip. Nature 2004, 431, 1081–1084. 10.1038/nature02921. 15510144

[ref41] Benítez J. L.; Hernández-cordero J.; Muhl S.; Mendoza D. Few Layers Graphene as Thermally Activated Optical Modulator in the Visible-near IR Spectral Range. Opt. Lett. 2016, 41, 167 10.1364/ol.41.000167. 26696185

[ref42] Lee M.; Heo M. H.; Lee H.; Lee H.-H.; Jeong H.; Kim Y.-W.; Shin J. Facile and eco-friendly extraction of cellulose nanocrystalsviaelectron beam irradiation followed by high-pressure homogenization. Green Chem. 2018, 20, 2596–2610. 10.1039/c8gc00577j.

[ref43] Koppolu R.; Abitbol T.; Kumar V.; Jaiswal A. K.; Swerin A.; Toivakka M. Continuous Roll-to-Roll Coating of Cellulose Nanocrystals onto Paperboard. Cellulose 2018, 25, 6055 10.1007/s10570-018-1958-1.

[ref44] TAPPI. Thickness (Caliper) of Paper, Paperboard, and Combined Board (TAPPI 411); Technical Association of Pulp and Paper Industry, 2015.

[ref45] Saleh B. E. A.; Teich M. C. Fundamentals of Photonics. Fundamentals of Photonics; Wiley Series in Pure and Applied Optics; John Wiley & Sons, Inc.: New York, USA, 1991.

[ref46] Lin N.; Dufresne A. Surface Chemistry, Morphological Analysis and Properties of Cellulose Nanocrystals with Gradiented Sulfation Degrees. Nanoscale 2014, 6, 5384–5393. 10.1039/c3nr06761k. 24706023

[ref47] Wang L.; Chen C.; Wang J.; Gardner D. J.; Tajvidi M. Cellulose Nanofibrils versus Cellulose Nanocrystals: Comparison of Performance in Flexible Multilayer Films for Packaging Applications. Food Packag. Shelf Life 2020, 23, 100464 10.1016/j.fpsl.2020.100464.

[ref48] Fang Z.; Zhu H.; Yuan Y.; Ha D.; Zhu S.; Preston C.; Chen Q.; Li Y.; Han X.; Lee S.; Chen G.; Li T.; Munday J.; Huang J.; Hu L. Novel Nanostructured Paper with Ultrahigh Transparency and Ultrahigh Haze for Solar Cells. Nano Lett. 2014, 14, 765–773. 10.1021/nl404101p. 24372201

[ref49] Aulin C.; Salazar-Alvarez G.; Lindström T. High Strength, Flexible and Transparent Nanofibrillated Cellulose-Nanoclay Biohybrid Films with Tunable Oxygen and Water Vapor Permeability. Nanoscale 2012, 4, 6622–6628. 10.1039/c2nr31726e. 22976562

[ref50] Xu X.; Zhou J.; Jiang L.; Lubineau G.; Ng T.; Ooi B. S.; Liao H.-Y.; Shen C.; Chen L.; Zhu J. Y. Highly Transparent, Low-Haze, Hybrid Cellulose Nanopaper as Electrodes for Flexible Electronics. Nanoscale 2016, 8, 12294–12306. 10.1039/c6nr02245f. 27270356

[ref51] Wu W.; Tassi N. G.; Zhu H.; Fang Z.; Hu L. Nanocellulose-Based Translucent Diffuser for Optoelectronic Device Applications with Dramatic Improvement of Light Coupling. ACS Appl. Mater. Interfaces 2015, 7, 26860–26864. 10.1021/acsami.5b09249. 26572592

[ref52] Kumar V.; Bollström R.; Yang A.; Chen Q.; Chen G.; Salminen P.; Bousfield D.; Toivakka M. Comparison of Nano- and Microfibrillated Cellulose Films. Cellulose 2014, 21, 3443–3456. 10.1007/s10570-014-0357-5.

