# Supplementary material for: Thermoresponsive Nanocellulose Films as an Optical Modulation Device: Proof-of-Concept
Source: ACS Appl Mater Interfaces. 2021 May 19;13(21):25346–56. doi: 10.1021/acsami.1c03541 (PMC8289189; doi:10.1021/acsami.1c03541)
Supplement: Supplementary file 1 — am1c03541_si_001.pdf [file am1c03541_si_001.pdf]

**Supporting Information**

# Thermoresponsive Nanocellulose Films as an Optical Modulation Device: Proof-of-Concept

*Aayush Kumar Jaiswal*[1], Ari Hokkanen[2], Vinay Kumar[1], Tapio Mäkelä[3], Ali Harlin[1], Hannes Orelma[1]*

[1]Biomass Processing and Products, VTT Technical Research Centre of Finland Ltd., Tietotie 4E, 02044 Espoo, Finland

[2]Microelectronics, VTT Technical Research Centre of Finland Ltd., Tietotie 3, 02044 Espoo, Finland

[3]Sensing and Integration, VTT Technical Research Centre of Finland Ltd., Tietotie 3, 02044 Espoo, Finland

Email: aayush.jaiswal@vtt.fi

KEYWORDS: nanocellulose, optical films, thermochromic, hybrid material, optical modulation

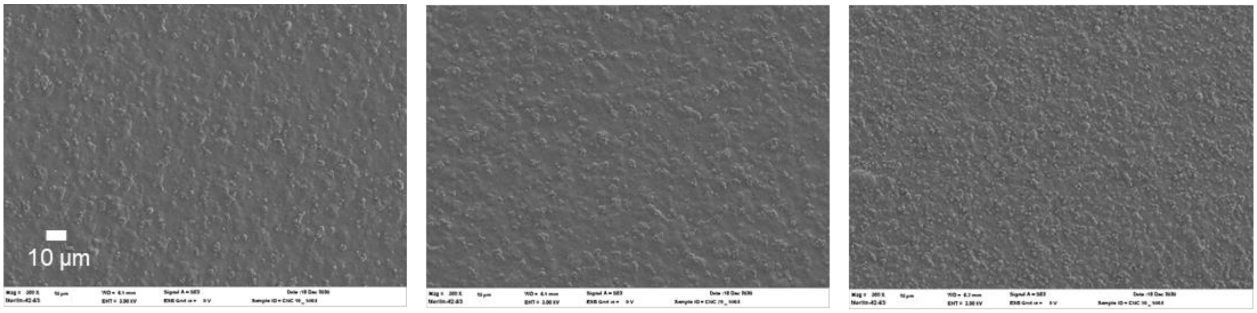


CNC10 CNC20 CNC30

Figure 1: SEM micrographs for the CNC film samples taken at 500X magnification.

Figure 1 shows the SEM micrographs for the CNF films samples which were not shown in the main text. These images supplement Figure 3 in the main text where only SEM micrographs of the CNC and CNC5 samples were shown to discuss the difference in film structure caused by the

introduction of thermochromic pigments.

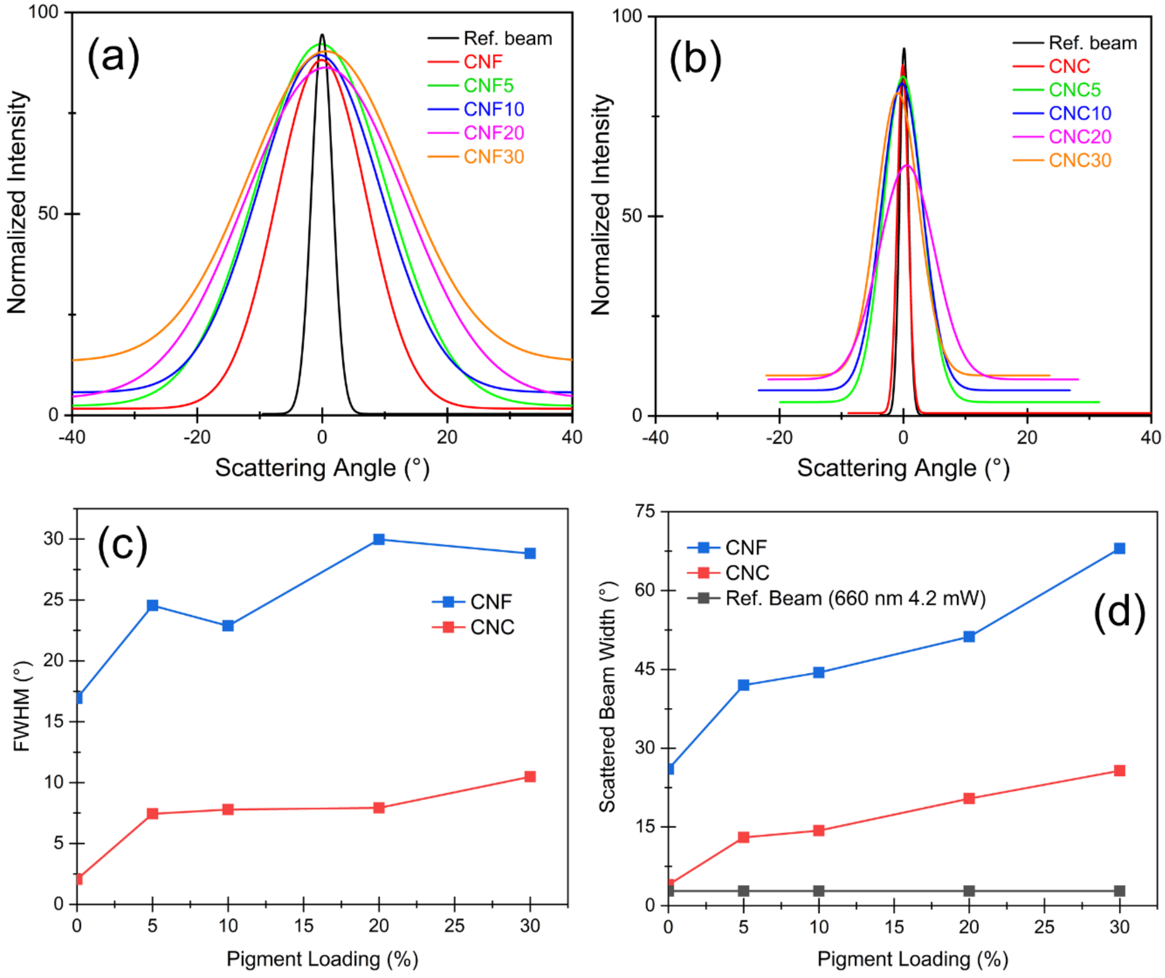


Figure 2 (a) and (b) show the Gaussian fits for the normalized intensity versus the scattering angle data. These plots supplement the results described in Figure 4 in the article. The intensity distribution of the forward scattered laser beam was first normalized and then fitted with the Gaussian function. The R-squared value for each fit was found to be greater than 0.92, indicating a good fit. The full width at half maximum (FWHM) values were calculated from the Gaussian fits and are shown in Figure 2 (c). Figure 2 (d) shows the scattered beam width measured during forward light scattering measurements as a function of pigment loading level for both CNF and CNC-based films. Typical for lasers, the reference laser beam was treated like a Gaussian beam and the beam width was calculated using the $1/e^2$ method, for both the reference 660 nm laser

beam and the beams scattered from the samples. The scattered beam width data can also be interpreted as the average forward scattering angle from the film samples.

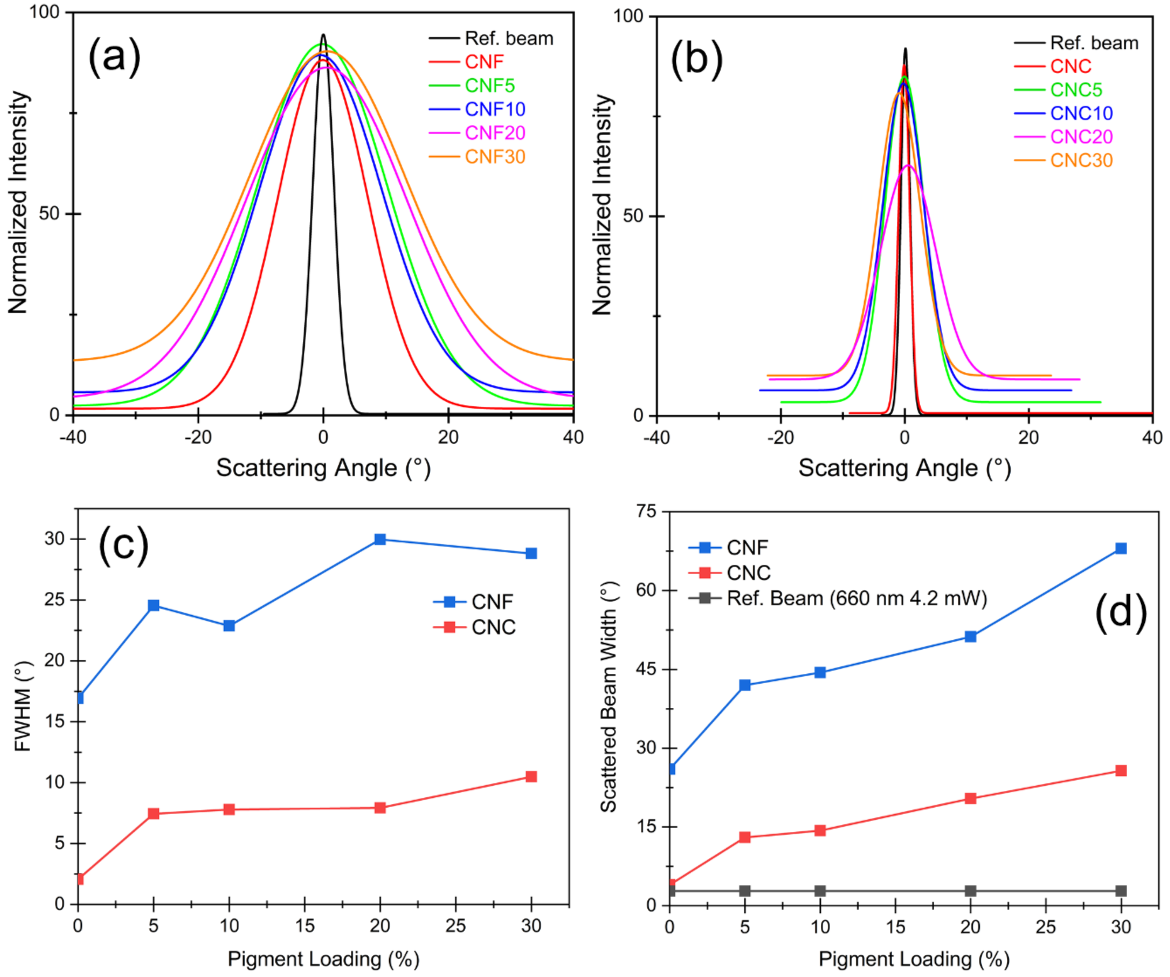


Figure 2. Gaussian fits for the normalized intensity versus scattering angle data during forward scattering measurement for (a) CNF and (b) CNC samples. Subfigure (c) shows the FWHM values for the Gaussian fits for both CNF and CNC samples. Subfigure (d) illustrates the scattering angle of the 660 nm laser beam through the sample films calculated using the $1/e^2$ method.

Figure 3 illustrates that the laser beam used during the modulation experiments was sufficient to cause spot heating and raise the film sample temperature locally above the transition temperature (31°C). Thermal imaging was used to confirm the temperature increase on the film sample.

Moreover, a transparent spot was clearly visible by the naked eye after the 6.3 mW laser beam was pulsed on the sample.

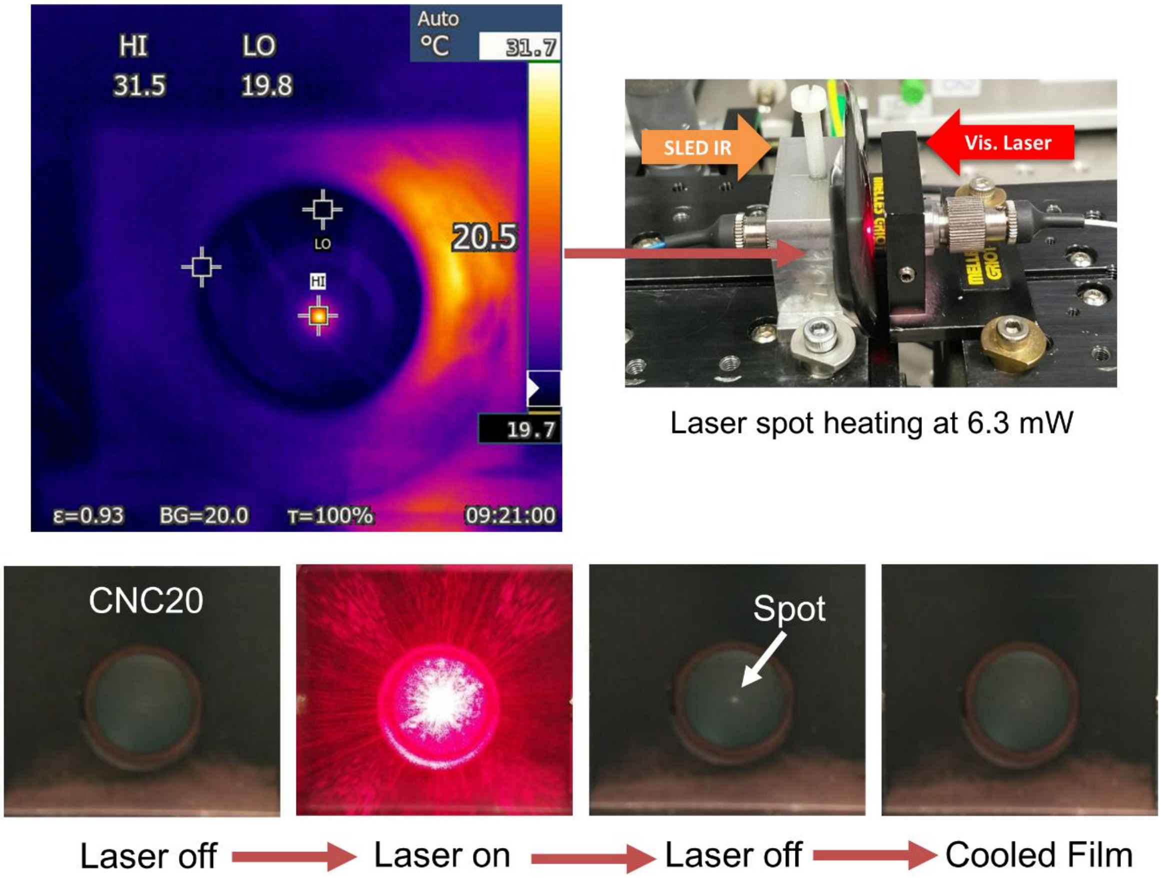


Figure 3. Examination of film sample temperature using a thermal imager during optical modulation experiments. A 660 nm red diode laser was used at 6.3 mW power to heat the measurement spot above the thermochromic transition temperature. The images at the bottom depict the reversible creation of a localized transparent spot due to the thermochromic effect in the CNC20 sample.

Figure 4 shows the modulation depths exhibited by CNF and CNC-based samples respectively under a 6.2 mW pulsed visible diode laser. This data supplements the results described in Figure 7 (c) and (d) in the main text where the modulation depth for only the CNF and CNC films, and the samples exhibiting the lowest and the highest performance was shown. Figure 4 here illustrates

the modulation depths for the remaining samples, along with the pure CNF and CNC films. The results have been shown separately to improve the readability of the article.

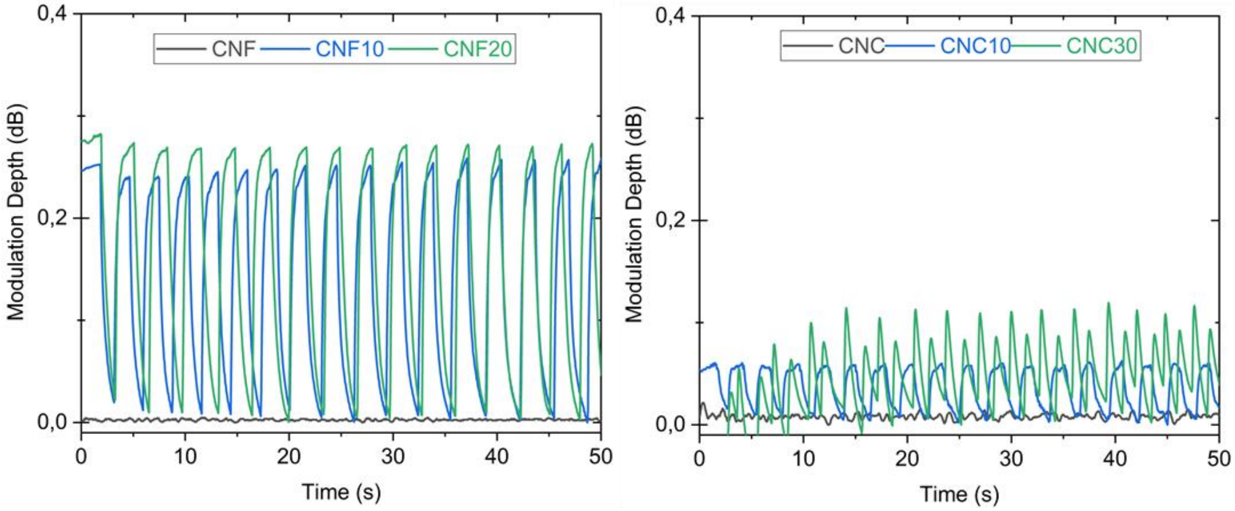


Figure 4. Continuous IR light modulation depths exhibited by CNF and CNC-based samples respectively under a 6.2 mW pulsed visible diode laser.
